# Metaplastic carcinoma of the breast with squamous differentiation: prognostic factors and multidisciplinary treatment

**DOI:** 10.1186/s12957-022-02656-5

**Published:** 2022-06-08

**Authors:** Ana Alicia Tejera Hernández, Víctor Manuel Vega Benítez, Marta Pavcovich Ruiz, Juan Ramón Hernández Hernández

**Affiliations:** 1 Faculty of Health Sciences, University of Las Palmas de Gran Canaria, Las Palmas, Spain; 2 General Surgery Department, Complejo Hospitalario Universitario Insular Materno-Infantil, Las Palmas, Spain; 3 Department of Pathology, Complejo Hospitalario Universitario Insular Materno-Infantil, Las Palmas, Spain

**Keywords:** Breast cancer, Metaplastic carcinoma, Squamous carcinoma, Survival

## Abstract

**Background:**

The objective of this study was to analyze the characteristics of patients diagnosed with metaplastic carcinoma of the breast with squamous differentiation and to identify the particular clinical and histological characteristics that need to be taken into account in this type of tumors.

**Case presentation:**

Retrospective observational study of two patients managed at our hospital between 2014 and 2020 (15 months mean follow-up), plus all cases published in the last 7 years (8 patients). Thus, a total of 10 cases were analyzed, all with less than 2 years mean global survival. Studied variables were: age, medical background, tumor size, axillary involvement, radiological characteristics, surgical approach, complementary treatments, histologic characteristics, and progression of the disease. In 50% of cases, the disease appeared as a palpable mass of rapid growth, associated with axillary infiltration; 80% of the tumors were triple negative; 30% of them progressed to distant metastatic disease in 30%.

**Conclusions:**

This unusual carcinoma requires a complex multidisciplinary treatment. Its prognosis is unfavorable due to its high local aggressiveness, with rapid progression and appearance of metastatic disease. The predominance of different histological components may determine the response to medical treatments.

## Background

Metaplastic breast carcinoma with squamous differentiation is an uncommon type of metaplastic carcinoma that accounts for less than 1% of breast carcinomas [1–3]. These tumors show an indolent course and may be associated to a preexistent benign lesion (adenomyoepithelioma, complex sclerosing lesions or fibrocystic disease) [4]. This heterogeneous type of tumors has been defined as an epithelial neoplasia of mixed squamous and glandular differentiation, with a widely variable response to oncologic therapy [1–3]. The high-grade presentation is the most aggressive one and the star-shaped infiltrating pattern is associated with a high probability of local relapse [4, 5]. In addition, due to their particular response to oncological treatment, eventual complex surgery is often needed. Here we present our experience with two cases diagnosed at our hospital, together with an analysis of all cases published in the last 7 years, especially regarding the clinical and histological characteristics that need to be considered in this type of tumor.

## Case presentation

Here, we present a retrospective observational study of patients with metaplastic breast carcinoma with squamous differentiation, who were managed at our hospital between 2014 and 2020, plus all cases published during the last 7 years. The following variables were described and analyzed: age, medical background, tumor size, axillary involvement, radiological characteristics, surgical technique used, complementary treatments, evolution of the disease and tumor histological type, histological grade, and immunohistochemical characteristics.

Only two patients were managed for this rare condition during the mentioned period, at our hospital (mean follow-up time of 15 months). Eight additional cases were identified in the literature, with similar general characteristics and with a mean overall survival time shorter than 2 years (13.6 months; one case was excluded from the calculation of mean survival because it was a pure in situ variant [6]).

### Case Nº1

**Fig. 1:**
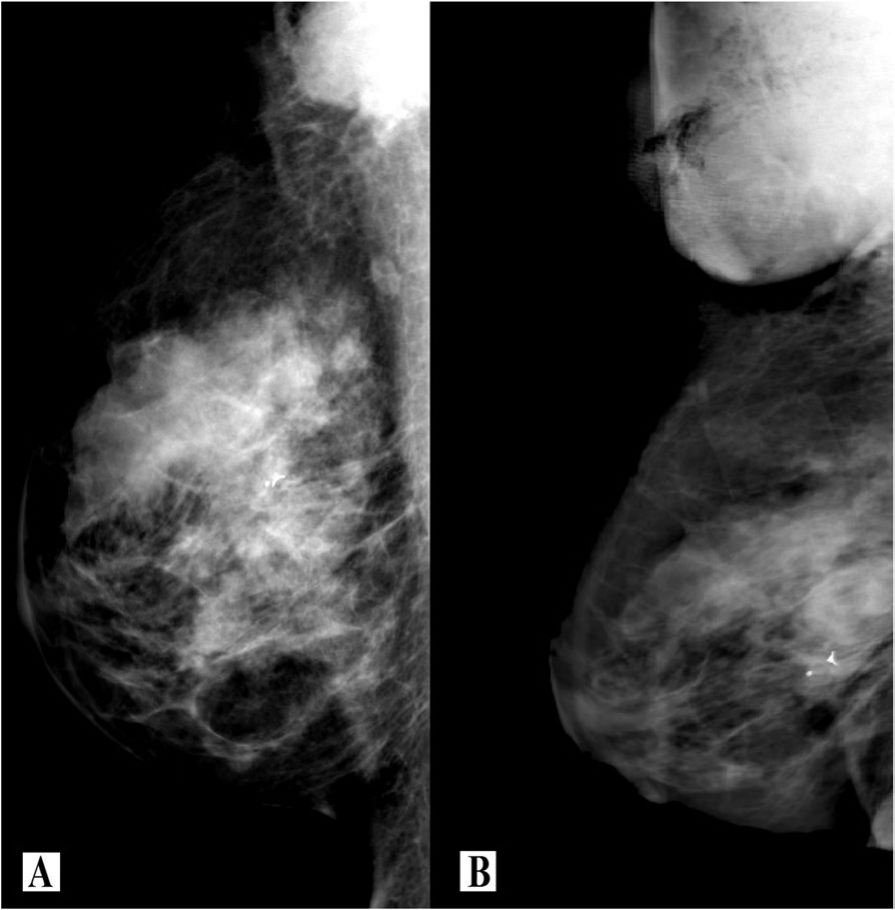
Case Nº1. Mammography study mid-lateral projection. **A** Diagnosis: large area of fibrocystic changes in the external upper quadrant of the breast (metallic marker); a 4-cm diameter mass in the armpit. **B** Evaluation of the response to chemotherapy: metallic markers fail to define underlying nodular lesion. Axillary progression with an important tumor mass of 10 cm

**Fig. 2:**
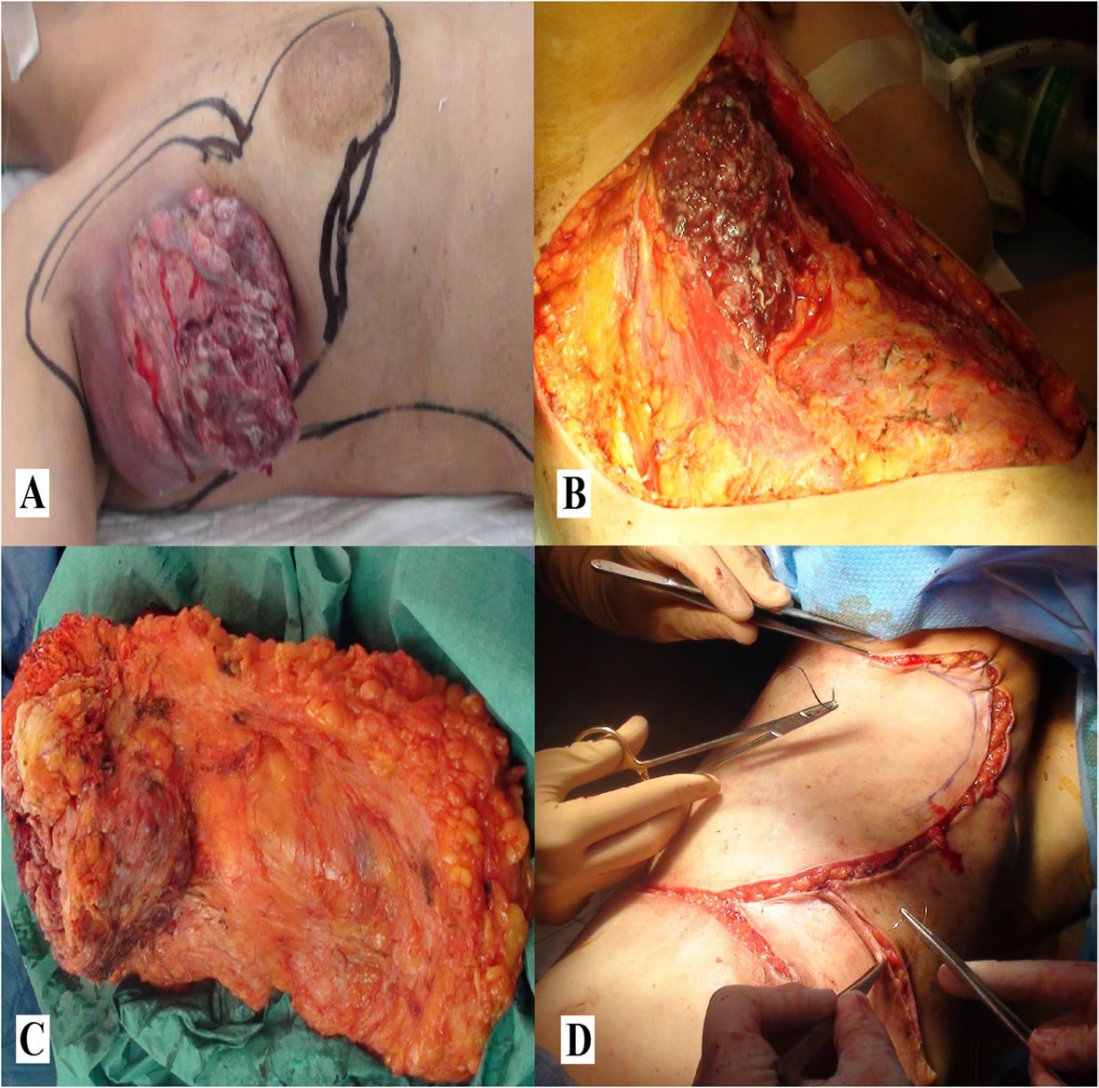
Case Nº1. surgery.** A** Ulcerated axillary tumoral mass of 12 cm. **B** Skin-sparing mastectomy and wide tumor resection, although leaving an irresectable tumor residue, which infiltrated the axillary vein, *latissimus dorsi* and right brachial plexus. **C** Macroscopic study of the surgical specimen: large ulcerated axillary tumor (12 × 10 cm), grayish-whitish in color with multiple necrotic and friable reddish areas, deep margin extensively involved. Dense breast parenchyma with multiple cysts; in the upper region, two adjacent zones of greater density of 3 and 2 cm that include metallic filament, free margins. **D** Close rotation myocutaneous flap of the *latissimus dorsi*

A 39-year-old female patient presented with a 5-cm nodule in the upper quadrants of the right breast, which had appeared 1 month before, plus inflammatory changes and palpable axillary adenopathy. She had undergone medical examination 6 years before, in a different healthcare center, due to fibrocystic disease. She provided three annual ultrasound studies that showed multiple cystic formations, parenchymal disruption, and ductal ectasia. She also provided the results of a biopsy study showing abundant epithelial and myoepithelial cellularity, fibrosis, cysts with apocrine metaplasia, and sclerosing adenosis. She had not received therapy. As a part of our examination, mammography and ultrasound studies were performed, where a large area of fibrocystic changes could be observed in the upper external quadrant of the right breast, as well as a 37-mm-diameter mass in the right armpit BI-RADS VI (Fig. 1A). Such findings were confirmed in a magnetic resonance study, which showed a 40-mm adenopathy plus metastatic involvement consisting of smaller surrounding adenopathies. Core needle biopsy revealed infiltrating carcinoma, probably of ductal origin, in the breast plus well-differentiated epidermoid carcinoma (p:63 positive) in the armpit, both were triple negative, ki 67: 60%, p:53 negative and ck 19 positive. Distant disease of stage T4N2M0 was ruled out. The tumor board decided that the patient be administered neoadjuvant chemotherapy consisting of four FEC cycles (5-fluorouracil-epirubicin-cyclophosphamide) and four docetaxel cycles. The treatment produced a 50% regression of the disease in the breast but a poor response in the armpit, where the disease actually progressed (Fig. 1B). Eventually, surgical management was scheduled (Fig. 2A).

The patient underwent skin-sparing mastectomy. Wide resection of the tumor was achieved, although an irresectable tumor residue remained, which infiltrated into the axillary vein, latissimus dorsi and right brachial plexus (Fig. 2B). The surgical specimen was collected for subsequent study (Fig. 2C). The defect was covered with a rotation myocutaneous flap of the latissimus dorsi. The donor zone was filled with flaps from the mastectomy (anterior part) and by dissecting the back until the midline and iliac crest (lower posterior part) (Fig. 2D). The surgical procedure was uneventful and the patient was discharged on day 3 postoperative. A histological study of the specimen revealed a high-grade 5-cm metaplastic ductal carcinoma with squamous differentiation, with axillary metastasis. In the breast, ductal predominance was found with associated high-grade intraductal carcinoma and free margins. In the armpit, a 12-cm ulcerated tumor mass was observed with a predominantly squamous pattern and extensive involvement of the deep margin. Adjuvant radiotherapy was initiated. However, 1 month later, ulcerations were observed in the armpit, which were related to the progression of the disease. Palliative chemotherapy was initiated. One month later, the patient reported low back pain and a D2–L1 metastatic lesion was found in bone scintigraphy. Later on, she presented dyspnea, multiple nodular lesions on both lungs and a metastatic lesion in the liver, all of which were managed with medical therapy until she died due to pulmonary embolism 11 months after diagnosis.

### Case Nº 2

**Fig. 3:**
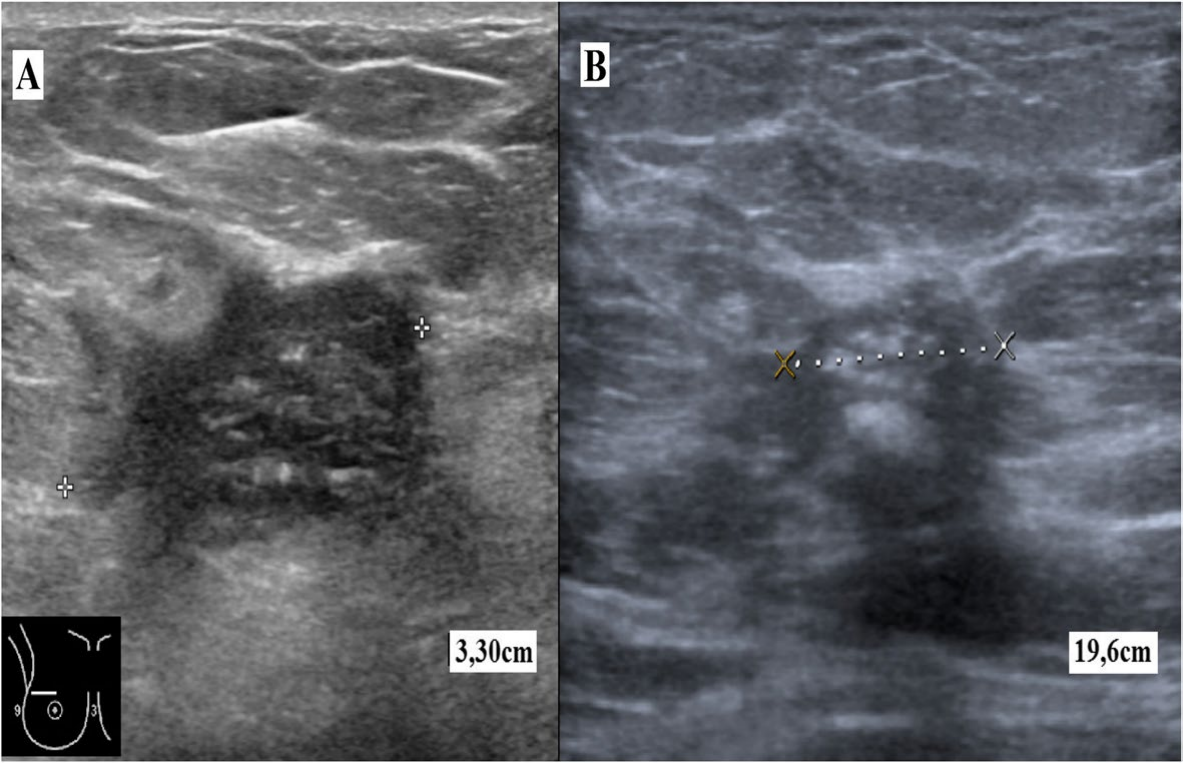
Case Nº2. Breast ultrasound study. **A** Hypoechoic solid nodule with internal microcalcifications; approximately 33 mm maximum diameter (BI-RADS V). **B** Diffuse microcalcifications of 2 cm size, the rest without alterations

A 57-year-old female patient presented with an 8-cm nodule in the upper quadrants plus 2-cm axillary adenopathy. She complained of a 1-month long pain in the right breast. Her medical record showed not events of interest. She underwent a mammography and an ultrasound study, which showed a hypoechoic solid nodule with internal microcalcifications, anfractuous edges, and 33 mm maximum diameter (BI-RADS V) (Fig. 3A). The ipsilateral axilla showed a ganglion with cortical thickening and infiltrative appearance. Core needle biopsy revealed metaplastic infiltrating ductal carcinoma with squamous differentiation, associated with high-grade intraductal carcinoma. Axillary lymph nodes were positive. An immunohistochemical study showed a triple negative, ki 67: 60%, p53 negative and ck 19-positive tumor. In an extension study, computed axial tomography and positron emission tomography showed two lesions indicative of liver metastasis on segments VI and II without signs of significant metabolic activity. The disease was in stage T2N1M1. The tumor board decided to administer neoadjuvant chemotherapy consisting of 4 cycles of anthracyclines and docetaxel. The treatment produced 50% disease regression in the breast (Fig. 3B) and the armpit, although with a poor response in the liver, where the disease progressed. The case was then re-evaluated and surgery was ruled out. The patient was treated with local–regional radiotherapy plus administration of two more lines of chemotherapy, in an attempt to maintain the distance disease under control. However, the response was poor and the patient died 19 months after the diagnosis due to severe liver failure.

Below we analyze the characteristics of our patients plus those reported in the literature (Table 1). The mean age was 52.5 years; 40% of them had pre-existing breast lesions (cases 2 [7], 5 [8], 6 [9], and 9); tumor size was large in all cases and axillary involvement was present in 50% of cases (cases 3 [10], 6, 7 [11], 9, and 10); 70% of patients presented radiological findings of a solid mass, four of them in association with a cystic component (cases 1[12], 5, 6, and 9). Less than a half of patients were diagnosed using core needle biopsy (cases 1, 4, 6, and 10) and three of them underwent an incomplete surgical resection (cases 7, 8[13], and 9). Six patients received chemotherapy: neoadjuvant for half of them (cases 1, 9, and 10) and post-surgery for the rest of them (cases 3, 6, and 7). Radiotherapy was used in half of the patients. As much as 80% of tumors were triple negative, 30% of them progressing to distant metastatic disease (liver, lungs, and brain).

## Discussion and conclusion

Metaplastic carcinoma with squamous differentiation of the breast is an uncommon neoplasia, which appears in women between 30 and 80 years of age, mostly postmenopausal. It may appear sporadically or derive from preexistent lesions involving breast glandular and tubular structures [12, 14, 15]. Clinically, a large palpable, well delimited, sometimes painful mass is observed, which has occasionally been reported as an incidental finding in an image diagnosis study [16, 17]. An ultrasound study may show an irregular hypo-echoic image; mammography may show a speculated mass or nodule, with increased density, poorly specific [17]. Core needle biopsy is not conclusive in most cases, since the whole histological architecture cannot be observed. The definitive diagnostic test is usually the histological study of the specimen [4, 14, 15].

In this series, we observe common characteristics in most cases. The onset of the disease presents as a palpable mass of rapid growth associated with axillary infiltration, with ages and radiological findings similar to those established in the literature. Almost all tumors were triple negative with a high ki 67 proliferation index, p: 53 negative and cytokeratin 19 positive reported in our center cases. In addition, although there was good response in the breast with chemotherapy treatment, progression of the metastatic disease was observed only in three cases. The usual chemotherapy schemes were used and radiotherapy was an adjuvant treatment that partially improved the aggression of the same.

Since these tumors are very rare, there is no consensus on their treatment. In general, the same guidelines than for other breast carcinomas are observed, although with some considerations. These are aggressive metaplastic tumors with high local relapse rates; thus, obtaining suitable margins in the surgical treatment is essential to prevent relapse [2, 18, 19]. Due to their size and poor response to chemotherapy, especially in its epithelial component, most cases require mastectomy and axillary surgery. When diagnosed early, a conventional conservative treatment might be possible [18, 20].

Like other metaplastic carcinomas, these tumors do not express hormonal receptors or Her 2, and have an unfavorable prognosis as compared with other triple-negative tumors, possibly due to their poor response to usual chemotherapeutic treatment [20, 21]. In an immunohistochemical study, marker p63 may be recognized in the squamous element, which facilitates classification; and they present positive cytokeratin [22, 23]. Radiotherapy is indicated and it is often used as an adjuvant treatment [24]. The tumor size, poor response to oncospecific treatment and local–regional relapse with subsequent distant disease are the main known prognostic factors to be considered [18, 20].

Metaplastic carcinoma of the breast with squamous differentiation is a rare neoplasia that requires a complex multidisciplinary treatment. Its prognosis is unfavorable due to its high local aggressiveness, rapid progression and development of metastatic disease. Predominance of different histological components of these tumors may determine their response to treatment and should be taken into account when a therapy is selected. Although our knowledge of metaplastic tumor variability is continuously growing, more studies are needed to develop new specific therapeutic strategies adjusted to the different types of patients.

**Table 1:** Reported single cases of Metaplastic  carcinoma with squamous differentiation of the breast (*n*=10)

**Case**	**Author,** **Year**	**Age**	**PL**	**Size** **(cm)**	**LNS**	**Radiological findings**	**Diagnostic**	**Surgery**	**CT**	**RT**	**IC**	**Distant metastatic **	**Survival (months)**
1	Graziano et al, 2015 [12]	59	No	Large mass	Negative	Solid/Cystic mass. No mic	CNB	R0	Neoadjuvant	NR	Triple negative	No	NR
2	Jagtap et al, 2015 [7]	45	Nipple retraction	Two Masses 9, 3	Negative	High density masses	Surgical specimen	R0	NR	NR	ER,PR negative Her 2 positive	No	NR
3	Nguyen et al, 2015 [10]	47	No	4	Positive	NR	Surgical specimen	R0	Adyuvant	Yes	ER positive PR, Her 2 negative	Lung and brain	NR
4	Arafah et al, 2016 [6]	73	No	Mic	Negative	Extensive calcifications	CNB	R0	No	No	Triple negative	No	11 years Pure insitu variant
5	Punzo et al, 2017 [8]	75	Inflamed cystic lesion	5	Negative	Solid/Cystic mass. Mic	Surgical specimen	R0	Patient refused	No	Triple negative p:63 positive	No	12
6	Chahdi et al, 2018 [9]	44	Cyst lesion	5	Positive	Solid/Cystic mass. Abscess	CNB	R0	Adyuvant	Yes	Triple negative	No	9
7	Goto et al, 2018 [11]	42	No	Large mass T4	Positive	Solid mass	Surgical specimen	R1	Adyuvant	Yes	Triple negative	No	17
8	Hardy et al, 2019 [13]	44	No	11	Negative	Solid necrotic mass	Surgical specimen	R1	Patient refused	No	Triple negativep:63 positive	No	NR
9	Current case 1	39	Fibrocystic disease	5	Positive	Solid/Cystic mass	Surgical specimen	R1	Neoadjuvant	Yes	Triple negativep:63 positive	Lungs and liver	11
10	Current case 2	57	No	8	Positive	Solid mass. Mic	CNB	No	Neoadjuvant	Yes	Triple negative	Liver	19

*PL* preexistent lesions, *LNS* Lymph node status, *CT* Chemotherapy, *RT* Radiotherapy, *IC* Immunohistochemical characteristics, *Mic* microcalcifications, *CNB* Core needle biopsy, *R0* Complete resection, *R1* Incomplete resection, *NR* Not reported, *ER* Estrogen receptor, *PR* Progesterone receptor

## Data Availability

The datasets used and/or analyzed during the current study were available from the corresponding author on reasonable request. These data were available in the clinical records system of our hospital.

## References

[1] Hashemi SM, Mahmoudi Shan S, Jahantigh M, Allahyari A (2017). Atypical breast adenosquamous carcinoma following acute myeloid leukemia in a middle-aged woman: a case report. Mol Clin Oncol.

[2] Pai T, Shet T, Desai S, Patil A, Nair N, Parmar V (2016). Impact of squamous differentiation in breast carcinoma. Int J Surg Pathol.

[3] Villalón-López JS, Souto-del Bosque R, Alonso-Briones MV, Trujillo-de Anda AP (2013). Carcinosarcoma of the breast a rare entity with fatal prognosis One case report. Cir Cir.

[4] Tse GM, Tan PH, Putti TC, Lui PC, Chaiwun B, Law BK (2006). Metaplastic carcinoma of the breast: a clinicopathological review. J Clin Pathol.

[5] Cakir A, Gönül II, Uluoğlu O (2012). Metaplastic breast carcinomas and their relationship with basal-like phenotype. Turk Patoloji Derg.

[6] Arafah M, Ginter PS, Taylor DC, Hoda SA (2016). Squamous cell carcinoma in situ of the breast: report of a case. Breast J.

[7] Jagtap SV, Bhosale SJ, Chougule PG, Dhawan SD, Shukla D (2015). Multicentric metaplastic breast carcinoma with squamous differentiation. J Clin Diagn Res.

[8] Punzo C, Fortarezza F, De Ruvo V, Minafra M, Laforgia R, Casamassina G (2017). Primitive squamous cell carcinoma of the breast (SCCB): case report of an uncommon variant of metaplastic carcinoma. G Chir.

[9] Chahdi H, Abdellah B (2018). Primary epidermoid carcinoma of the breast mimicking an abscess. Pan Afr Med J.

[10] Nguyen DN, Kawamoto S, Cimino-Mathews A, Illei PB, Rosenthal DL, VandenBussche CJ (2015). Metastatic metaplastic breast carcinoma mimicking pulmonary squamous cell carcinoma on fine-needle aspiration. Diagn Cytopathol.

[11] Goto Y, Yoshida T, Kimura M (2018). Higher efficacy and complete response with administration of eribulin for recurrent squamous cell breast carcinoma: A case report. Mol Clin Oncol.

[12] Graziano L, Filho PG, Bitencourt AG, Soto DB, Hiro A (2015). Metaplastic squamous cell carcinoma of the breast: a case report and literature review. Rev Assoc Med Bras.

[13] Hardy BM, Cortina CS, Javidiparsijani S, Ghai R, Madrigrano A (2019). Hypercalcemia in metaplastic squamous cell carcinoma of the breast. Am J Case Rep.

[14] Gobbi H, Simpson JF, Jensen RA, Olson SJ, Page DL (2003). Metaplastic spindle cell breast tumours arising within papillomas, complex sclerosing lesions, and nipple adenomas. Mod Patho.

[15] Denley H, Pinder SE, Tan PH, Sim CS, Brown R, Barker T (2000). Metaplastic carcinoma of the breast arising within complex sclerosing lesion: a report of five cases. Histopathology.

[16] Barnes PJ, Boutilier R, Chiasson D, Rayson D (2005). Metaplastic breast carcinoma: clinical-pathologic characteristics and HER2/neu expression. Breast Cancer Res Treat.

[17] Choi BB, Shu KS (2012). Metaplastic carcinoma of the breast: multimodality imaging and histopathologic assessment. Acta Radiol.

[18] Leyrer CM, Berriochoa CA, Agrawal S, Donaldson A, Calhoun BC, Shah C (2017). Predictive factors on outcomes in metaplastic breast cancer. Breast Cancer Res Treat.

[19] Yadav S, Yadav D, Zakalik D (2017). Squamous cell carcinoma of the breast in the United States: incidence, demographics, tumor characteristics, and survival. Breast Cancer Res Treat.

[20] Aydiner A, Sen F, Tambas M, Ciftci R, Eralp Y, Saip P (2015). Metaplastic breast carcinoma versus triple-negative breast cancer: survival and response to treatment. Medicine (Baltimore).

[21] Aggarwal G, Reid MD, Sharma S (2012). Metaplastic variant of invasive micropapillary breast carcinoma: a unique triple negative phenotype. Int J Surg Pathol.

[22] Tse GM, Tan PH, Chaiwun B, Putti TC, Lui PC, Tsang AK (2006). p63 is useful in the diagnosis of mammary metaplastic carcinomas. Pathology.

[23] Geyer FC, Lambros MB, Natrajan R, Mehta R, Mackay A, Savage K (2010). Genomic and immunohistochemical analysis of adenosquamous carcinoma of the breast. Mod Pathol.

[24] Tseng WH, Martinez SR (2011). Metaplastic breast cancer: to radiate or not to radiate?. Ann Surg Oncol.

